# Yttrium-90 Radioembolization for Colorectal Cancer Liver Metastases: A Single Institution Experience

**DOI:** 10.1155/2011/571261

**Published:** 2011-03-20

**Authors:** Gary W. Nace, Jennifer L. Steel, Nikhil Amesur, Albert Zajko, Bryon E. Nastasi, Judith Joyce, Michael Sheetz, T. Clark Gamblin

**Affiliations:** ^1^Department of Surgery, University of Pittsburgh School of Medicine, Pittsburgh, PA 15213, USA; ^2^Department of Psychiatry, University of Pittsburgh School of Medicine, Pittsburgh, PA 15213, USA; ^3^Department of Radiology, University of Pittsburgh School of Medicine, Pittsburgh, PA 15213, USA; ^4^Department of Surgery, Medical College of Wisconsin, Milwaukee, WI 53226-3596, USA

## Abstract

*Purpose*. We sought to evaluate our experience using yttrium-90 (^90^Y) resin microsphere hepatic radioembolization as salvage therapy for liver-dominant metastatic colorectal cancer (mCRC). *Methods*. A retrospective review of consecutive patients with unresectable mCRC who were treated with ^90^Y after failing first and second line systemic chemotherapy. Demographics, treatment dose, biochemical and radiographic response, toxicities, and survival were examined. *Results*. Fifty-one patients underwent ^90^Y treatments of which 69% were male. All patients had previously undergone extensive chemotherapy, 31% had undergone previous liver-directed therapy and 24% had a prior liver resection. Using RECIST criteria, either stable disease or a partial response was seen in 77% of patients. Overall median survival from the time of first ^90^Y treatment was 10.2 months (95% CI = 7.5–13.0). The absence of extrahepatic disease at the time of treatment with ^90^Y was associated with an improved survival, median survival of 17.0 months (95% CI = 6.4–27.6), compared to those with extrahepatic disease at the time of treatment with ^90^Y, 6.7 months (95% CI = 2.7–10.6 Conclusion: ^90^Y therapy is a safe locoregional therapy that provides an important therapeutic option to patients who have failed first and second line chemotherapy and have adequate liver function and performance status.

## 1. Introduction

Colorectal carcinoma, estimated to occur at an incidence of 148,810 cases in the USA in 2008 causing 49,960 deaths, is the major contributor of metastatic liver tumors [1]. Hepatic metastases are present in 15–25% of patients at presentation, and an additional 25–50% will develop liver metastases within 3 years following resection of the primary tumor [2, 3]. In approximately half of these patients, metastatic disease is confined to the liver, and 20% of all patients who die of metastatic colorectal cancer have metastases limited to the liver. Hepatic resection for colorectal liver metastases has become the standard of care, and currently remains the only potentially curative therapy. Unfortunately, curative resection is possible in less than 25% of those patients with metastases to the liver.

Currently, the armamentarium against unresectable liver tumors is composed of a number of liver-directed therapies aimed at reducing the hepatic tumor burden. Yttrium-90 (^90^Y) bound microspheres are an emerging tool for the treatment of primary and metastatic liver cancer that has had promising results. The use of whole-liver external beam radiation therapy for hepatic malignancies has been limited secondary to the relative intolerance of normal liver parenchyma to the dose of radiation necessary to have a response in neoplastic tissue. It has long been known that hepatic parenchyma is largely supplied by the portal system, but hepatic neoplasms are primarily supplied by the arterial system [4]. Therefore, therapy directed into the hepatic arterial system is preferentially targeted to the neoplasm with relative sparing of the normal parenchyma allowing for substantially higher doses of radiation or chemotherapeutic agents to be administered to the liver tumor tissue. Selective internal radiation therapy (SIRT), using ^90^Y microspheres delivered into the hepatic arterial system, takes advantage of the heterogeneity in blood supply between neoplastic and parenchymal tissue allowing for localized high-dose radiation therapy to be delivered to intrahepatic tumors. In addition to providing localized radiation therapy, the microspheres may also serve to provide an embolic component to the therapy leading to tumor ischemia. Pathologic examination of explants has shown that ^90^Y microspheres are dispersed preferentially to the periphery of neoplastic tissue [5].

^90^Y is a pure beta-emitting isotope with a maximal energy of 2.27 MeV and average energy of 0.94 MeV. The maximum range of emission in tissue is 11 mm with mean range of 2.5 mm, allowing tissue only in close proximity to the embolized microspheres to be treated. The half-life of ^90^Y is 64.1 hours with 94% of radiation delivered in 11 days [6]. Currently in the U.S. market, there are two commercially available microspheres that are irreversibly bound to^ 90^Y; the microspheres are composed of either resin or glass. Both are biocompatible beads with an average diameter of 20–40 *μ*m that are permanently implanted in the liver via embolization through the hepatic artery. The resin microspheres received U.S. Food and Drug Administration (FDA) approval in 2002 for unresectable liver metastases from primary colorectal cancer with adjuvant intrahepatic chemotherapy using floxuridine (FUDR). Glass microspheres were granted humanitarian device exemption (HDE) in 1999 for radiation treatment or as a neoadjuvant to surgery or transplantation in patients with unresectable hepatocellular carcinoma [6, 7].

Several studies have previously shown that ^90^Y microsphere radioembolization produce adequate response rates in colorectal cancer liver metastases with an acceptable toxicity profile [8–19]. However, many of these earlier experiences with ^90^Y occurred prior to widespread availability of the newer chemotherapeutic agents and regimens. With the addition of biological agents, such as bevacizumab and cetuximab, to chemotherapy regimens incorporating irinotecan and oxaliplatin the median survival rates and response rates improved in patients with mCRC [20, 21]. The effectiveness of ^90^Y in patients after failing the latest systemic chemotherapy has yet to be fully evaluated. We sought to retrospectively evaluate our single-institution experience treating patients with liver-dominant mCRC in the salvage setting with ^90^Y resin microsphere radioembolization. 

## 2. Material and Methods

### 2.1. Patient Selection, Workup, and Treatment

All patients in this series were treated consecutively with ^90^Y radioembolization between August 2002 and May 2008 for liver-dominant mCRC at the University of Pittsburgh Medical Center. Patients eligible to receive ^90^Y were not candidates for hepatic resection or ablation, had progressive disease after first and second line chemotherapy, had Eastern Conference Oncology Group (ECOG) performance status of 0-1, had adequate hepatic function (serum total bilirubin <2.0), and had adequate renal and hematologic function. Progressive disease was defined as increase in tumor burden by radiographic volume or number of metastases. Both intra- and extrahepatic tumors were evaluated for progression, although intrahepatic progression was the determinate for ^90^Y treatment candidacy. Patients that were considered for ^90^Y treatment had either exhausted or refused standard chemotherapy regimens. Patients with extrahepatic metastases were treated only if the tumor burden outside the liver was <10% of total tumor burden and chemotherapy options were not available. Data was recorded via an Institutional Review Board approved protocol.

The technical details and dosimetry of the process have previously been described [22, 23]. However, we chose to use a modified partition model for the calculation of the ^90^Y microsphere activity to be administered to the patient, similar to the methodology used for ^90^Y glass microspheres. The prescribed activity was calculated to deliver 50 Gy to the targeted liver tissue, assuming a uniform distribution of microspheres in the normal liver parenchyma and tumors, with no correction for any activity shunted to the lungs:

(1) $$\begin{array}{cc} & \text{Activity}\;\left(\text{GBq}\right)=\text{Dose}\;\left(\text{Gy}\right)\times \text{Mass}\;\left(\text{kg}\right)/50. \end{array}$$

The liver mass is determined from the target liver volume obtained from CT(cm3) × 0.00103 kg/cm^3^. This methodology was thought to more accurately estimate the radiation dose to the normal liver parenchyma for lobar and segmental treatments than the BSA or Empirical dosing methodologies. However, because of the nonuniform distribution of microspheres in the tumor and normal liver tissue, a proportionally larger radiation dose will be delivered to the tumor tissue and less to the normal liver [22, 23]. Only resin microspheres were used to treat patients in this study. Prior to ^90^Y administration, all patients had a selective visceral angiogram, technetium-99m-labeled macroaggregated albumin (^99m^Tc-MAA) study, and a baseline CT or PET/CT.

The selective visceral angiogram allows for definitive assessment of the arterial anatomy and possible embolization of vessels that may lead to extrahepatic ^90^Y exposure. The ^99m^Tc-MAA study allows pulmonary shunting to be evaluated. The use of ^90^Y radioembolization is avoided if there is any uncorrectable extrahepatic shunting to the gastrointestinal tract, or >0.6 GBq (corresponding to a lung dose of 30 Gy) is shunted to the lungs.

^90^Y was administered via unilobar treatments. When bilobar disease was present, the lobes were treated sequentially with approximately a one-month interval. Some patients had multiple treatments to the same lobe. The determination to treat the same lobe repetitively was made by evaluating the performance status, liver function, and extent of extrahepatic disease. However, the determination to retreat patients was based solely on progression of disease as assessed via CT. Early in our experience 17 patients had received floxuridine (FUDR) (5 mg/kg) infused via the hepatic artery just prior to instillation of ^90^Y microspheres. This was stopped due to concerns of FUDR-induced biliary sclerosis seen in prior hepatic artery infusion pump therapy cases, but not in these particular patients. After treatment, patients were observed in the hospital overnight and patients were discharged home the next day with oral narcotics and antiemetics.

### 2.2. Patient Followup and Evaluation

Toxicity data was reviewed from hospital records and laboratory data. Laboratory data was assessed using National Cancer Institute's Common Terminology Criteria for Adverse Events v3.0. Patients were followed with weekly laboratory data and routine office followup. A CEA response was defined as a ≥50% reduction in posttreatment value when compared to baseline measurements at the time of initial ^90^Y treatment. Followup imaging was performed every 3 months using either triphasic CTs or PET/CTs. The radiologic response was graded using the RECIST criteria [24]. In brief, the sum of the longest diameter of the five largest hepatic lesions was measured on baseline and follow-up imaging. A complete response was defined as disappearance of all target lesions, partial response was at least a 30% decrease in the overall diameter, and progressive disease was at least a 20% increase in diameter. Stable disease were those cases between partial response and progressive disease. Follow-up imaging from 30–180 days after the initial treatment were used for data analysis.

### 2.3. Statistics

Data were entered and verified using the Statistical Package for Social Sciences (SPSS version 16) for Windows (SPSS Inc., Chicago, IL). Kaplan-Meier survival analyses using log-rank methods were used to estimate overall survival of the entire sample as well as to test differences in survival between groups. Mean and median survival was reported (with 95% confidence intervals). Variables studied in the univariate analysis included gender, age (>50 and <50 years), presence of extrahepatic disease, prior liver-directed therapy, chemotherapy failure, number of treatments (<2 or ≥2), whether FUDR was given concurrently with^90^Y, CEA response, radiologic response, and total amount of radiation delivered prior to embolization (< or ≥80% of prescribed dose). Survival was calculated from the time of first ^90^Y treatment to the time of death.

## 3. Results

### 3.1. Demographics, Treatment Regimen, and Tumor Characteristics

Fifty-one patients were treated a total of 90 times with ^90^Y microspheres. The median age of patients treated was 64 years (range 37–83) (Table 1). All patients had undergone extensive chemotherapy with 33 (73%) patients receiving either bevacizumab or cetuximab, and 9 (20%) patients receiving both. Fourteen (31%) patients had received prior capecitabine. Previous liver-directed therapy was performed in 16 (31%) patients. Liver-directed treatment included radiofrequency ablation in 11 patients, and previous hepatic artery chemoinfusion in 5 patients. Liver resections were performed in 12 (23%) patients prior to ^90^Y therapy.

Although all patients had liver-dominant metastatic disease, a substantial number had radiographically demonstrable extrahepatic metastases. The presence of extrahepatic disease was known in 28 (58%) patients at the time of ^90^Y treatment. The sites of extrahepatic disease included pulmonary nodules (*n* = 14), portocaval or retroperitoneal lymphadenopathy (*n* = 13), anastomotic recurrence or unresected primary colorectal tumor (*n* = 5), peritoneal disease (*n* = 4), bone metastases (*n* = 1), and adrenal metastases (*n* = 1).

The median time from the diagnosis of hepatic metastases to the first ^90^Y treatment was 23.2 months (range, 1.3–99.9 months). One treatment was administered to 20 (39%) patients, 2 treatments to 27 (53%) patients, and 4 treatments to 4 (8%) patients (Table 2). The median lung shunt was 3.3% (range, 0.4–11.5%). No patients were excluded from treatment due to either an unacceptable level of pulmonary shunting or uncorrectable shunting to the extrahepatic gastrointestinal tract. The median dose administered to the target lobe per treatment was 44.4 Gy. The median prescribed activity of ^90^Y administered was 1.10 GBq versus the median activity actually delivered of 0.89 GBq. FUDR was given prior to ^90^Y in 17 (33%) patients, all of which were treated at the start of the study period. In 67 (74%) treatments ≥80% of prescribed dose was administered. All of those with <80% of the prescribed dose received had the treatment terminated due to the stagnation of flow secondary to the embolic process. There was no survival differences found when the actual dose administered was analyzed. However, patients who had one or more of the treatments terminated before the target dose was reached had a trend (*P* = .12) towards improved survival. Those who had stagnation of flow prior to reaching the target dose had a median survival of 19.1 months (95% CI = 8.7–29.5) compared to 6.7 months (95% Cl = 2.3–11.0). The median percentage of the prescribed dose administered in all treatments was 92%. There was no relationship between premature embolization during treatment and concurrent administration of FUDR.

### 3.2. CEA Response

In 41 patients, serial CEA levels were available for review. A CEA response (≥50% decreases in CEA from baseline) was seen in 17 (41%) patients (Table 3). For those who had a CEA response, the median value was 28.5% (range 1–42%) of the baseline before ^90^Y value. The CEA nadir was measured at a median of 62 (range 25–139) days after ^90^Y treatment.

### 3.3. Radiologic Response

Although all patients had baseline and follow-up imaging obtained, only 31 patients had imaging available for review. This is a reflection of our tertiary care center and many patients received scans closer to home, and these were not available for retrospective review. Extensive efforts were undertaken to obtain imaging that patients received outside institutions but many radiologic studies were not available for retrospective interpretation. Imaging was evaluated for a response to treatments using the RECIST criteria reviewing CT scans from 1–6 months after treatment. Although, many patients had PET/CT available towards the end of the series they were not obtained consistently enough for analysis in patients early in the series. No patients had a complete radiologic response. A partial response (PR) was observed in 4 (13%) patients, stable disease was seen in 20 (64%), and progressive disease (PD) in 7 (23%). Of the 7 patients that had PD during the 1–6 months follow-up period, 3 patients had met criteria within 60 days, 3 had developed PD from 61–120 days, and 1 after 121 days. Of the 4 patients with PR, 2 had met criteria within 60 days and 2 within 61–120 days.

### 3.4. Survival Analysis

Survival data was acquired from the electronic medical record and a search of the Social Security Death Index. Thirty-eight (74%) patients died during the follow-up period. The median survival with 95% confidence interval and *P-*values were calculated for several variables to identify differences in survival between groups. The variables for the univariate analyses included gender, age, presence of extrahepatic disease, prior liver-directed therapy, chemotherapeutic agents failed, number of treatments (< or ≥2), concurrent administration of FUDR, CEA response, radiologic response, and early termination due to embolic process. Of the tested variables, the only variable that showed a significant difference in survival was the use of cetuximab prior to ^90^Y treatments. 

**Figure 1:**
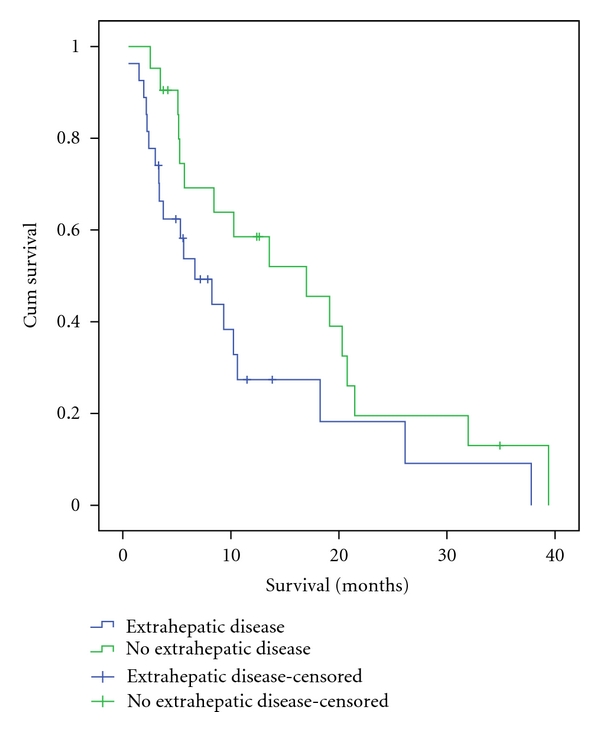
Kaplan-Meier survival curve comparing those with extrahepatic disease at the time of treatment versus those with disease localized to liver. The estimated median survival was 17.0 months for those without extrahepatic disease compared to 6.7 months. This difference was found not to be significant when using log-rank analysis, *P-*value of  .07.

**Figure 2:**
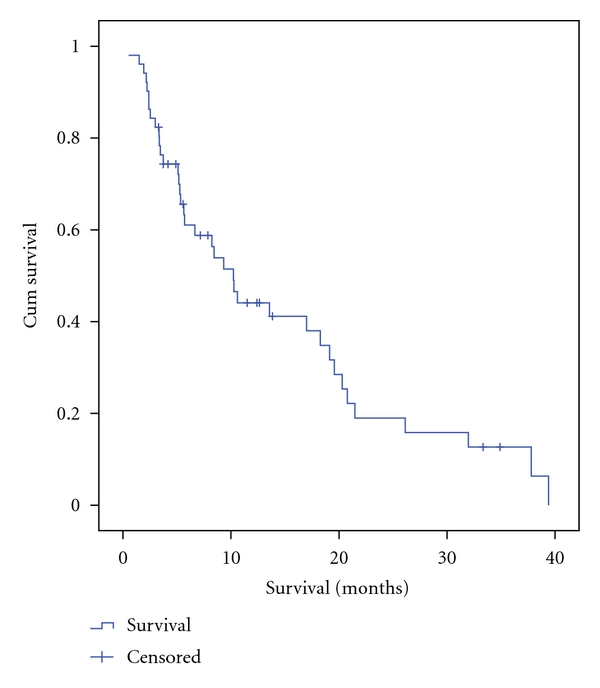
Kaplan-Meier survival curve for all patients (*n* = 51) treated with ^90^Y radioembolization. Survival was calculated from time of the first treatment with^90^Y. Median survival was estimated to be 10.2 months. The 95% confidence-interval is 7.5–13.0 months.

The overall median survival was 10.2 months (95% CI = 7.5–13.0) for the entire cohort. The overall mean survival was 14.4 months (95% CI = 10.6–18.1). The Kaplan-Meier survival curve including all treated patients is shown in Figure 2. When the presence or lack of extrahepatic disease was analyzed for an association with survival, there was a trend toward improved survival in the absence of extrahepatic disease. The median survival was 6.7 months with extrahepatic disease (95% CI = 2.7–10.6) compared to 17.0 months for those without extrahepatic disease (95% CI = 6.4–27.6) (Figure 1). Those who had received cetuximab had a significantly decreased median survival (*P* = .001) (5.1 months; 95% CI = 2.6–7.5) compared to patients that had not received cetuximab prior to ^90^Y (18.3 months; 95% CI = 6.5–30.0). A significant survival difference was also observed in those that had previously failed cetuximab and bevacizumab, but not those who failed only bevacizumab (Table 4). Although a larger proportion of patients received biological agents during the final years of the study period, analysis comparing the era of 2002–2004 versus 2005–2008 revealed no significant difference in survival between the two eras. 

**Figure 3:**
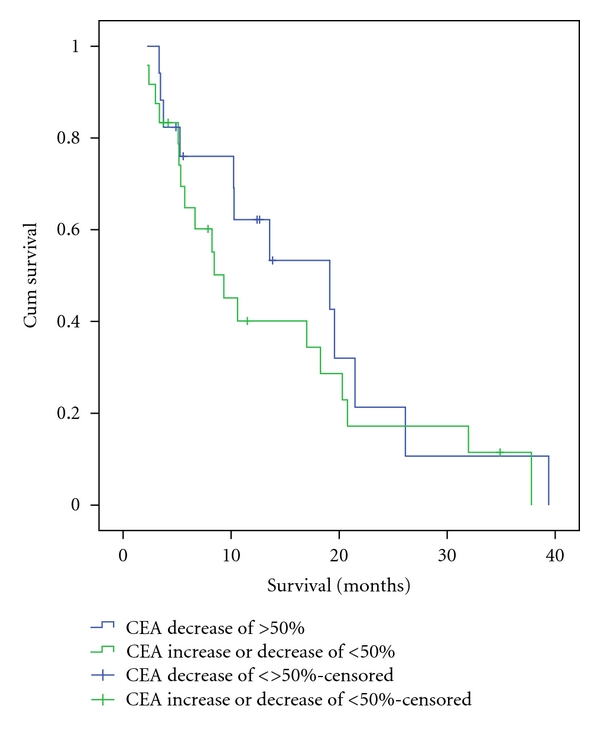
Kaplan-Meier survival curve comparing those with CEA response, as indicated by a decrease of ≥50% from baseline, to those who did not have a CEA response posttreatment. The estimated median survival was 19.1 months for those with a CEA response compared to 9.3 months. This difference was not found to be significant when using log-rank analysis *P-*value of  .36.

CEA response was not associated with a significant improvement in survival (Figure 3). Those with a CEA decrease of ≥50% from baseline had a 19.1-month (95% CI = 6.4–31.9) median survival compared to 9.3 months (95% CI = 6.0–12.7) in those with less than a 50% decrease in CEA. When a radiologic response was analyzed for a survival advantage, there was not a significant association with survival observed (*P* = .22). Patients with a partial response had the longest median survival (21.5 months; 95% CI = 13.8–29.2) when compared to those who had progressive disease (13.6 months; 95% CI = 7.4–19.8) and those who had a stable radiological response (9.3 months; 95% CI = 6.9–11.8 months). The unexpected results of a longer median survival in those with progressive disease compared to stable disease is likely due to the small sample size and lack of events per group (i.e., 43% censored in progressive disease).

### 3.5. Safety and Toxicity

Patient medical records were carefully reviewed to investigate patient complaints after treatment. The clinical toxicity profile was acceptable with fatigue, abdominal pain, and nausea being the most common subjective complaints documented; occurring in 22, 16, and 12% of patients, respectively. Complaints were minor, grade 1 or 2, and self-limiting generally resolving within one to two weeks after treatment. Three patients required hospital readmission within 30 days. Reasons for readmission included an upper GI bleed related to esophageal varices 4 days after treatment, unresolved abdominal pain and need for intravenous narcotics on postprocedure day 1, and the development of symptomatic brain metastases. One patient developed a complication at the time of the procedure developing ventricular tachycardia requiring ACLS and subsequent emergent cardiac catheterization. The posttreatment hepatic toxicity was assessed and found to be relatively mild. No patients had fulminant hepatic failure after treatment. Serial posttreatment bilirubin levels were available for review in 49 patients. Of the 47 patients who had a normal starting bilirubin levels, a grade 2 bilirubin toxicity was seen in 5 patients acutely (0–30 days) and 4 patients late (31–90 days). Late grade 3 or 4 toxicity was seen in two patients. The grade 4 toxicity was related to a biliary stricture and resolved with ERCP and stenting. No patients developed posttreatment gastric or duodenal ulceration, although all patients were placed on proton pump inhibitors prophylactically.

## 4. Discussion

Although the survival of patients with mCRC has been substantially extended with modern chemotherapy, the eventual progression of disease without a surgical cure is inevitable. Unfortunately, a surgical cure is only a viable option in the minority of patients. In this retrospective study we evaluated the efficacy and safety of using ^90^Y hepatic radioembolization in the salvage setting for advanced liver-dominant mCRC. Our cohort of patients consisted of a group that was highly pretreated with the current, most effective chemotherapeutic regimens. The median and mean survival of 10.2 and 14.4 months, respectively, after failure of all current treatment options is notable. The median survival is similar to that previously reported by others. Kennedy et al. had previously published their series of 208 patients treated with either whole liver or lobar ^90^Y for salvage therapy of unresectable mCRC. The median survival in this study was 10.5 months for those with treatment response and 4.5 months in those without a response to treatment [12]. Jakobs et al. had reported on a cohort of 41 patients that received mainly whole-liver ^90^Y that for liver-dominant mCRC with a median survival 10.5 months. One notable difference between the cohort reported here and that of Jakobs et al. is that in this cohort the presence of extrahepatic disease was 58.3% versus 17%, possibly signifying that our patients had more advanced disease. Sato et al. have also reported their two institution experiences of 137 patients with an assortment of primary malignancies treated with ^90^Y. Their cohort also contained 51 patients with mCRC, and they reported a median survival of 15.2 months in their subgroup analysis of colorectal cancer patients [15, 19].

There was significantly decreased survival observed in those who had previously failed cetuximab or both cetuximab and bevacizumab. A trend in decreased survival was seen in those who had previously failed bevacizumab. This result seems to suggest that benefit may be more limited in those who had previously failed biological chemotherapeutics. However, further validation of these findings is needed. Although unclear at this point, possible reasons for this difference could be that more aggressive tumor biology was selected or that patients were treated later in their disease course.

Surprisingly patients who had one or more of their treatments terminated with <80% of the prescribed dose administered had a trend towards improved survival. There is likely a range of effective dose of ^90^Y that elicits a tumor response, with patients who receive less than the prescribed dose also demonstrating a response to treatment most likely related to the completeness of the embolization. However, this observation requires further evaluation.

A trend towards improved survival was seen in those without extrahepatic disease at the time of treatment when compared to those with extrahepatic disease. The median survival was 17.0 months versus 6.7 months. Although this difference was not significant, our survival is comparable to the median survival of 17.5 months without extrahepatic disease and 6.9 months with extrahepatic disease that was previously reported [25]. The treatment of patients with widespread systemic disease has been an exclusion criteria for most treatments using locoregional therapies. In those who have already failed the first lines of the standard chemotherapeutic regimens, even in the face of a significant response of the liver metastases, they ultimately have progression of extrahepatic disease which will limit the survival. However, as seen in this study and others, the toxicity profile is acceptable in those with limited systemic disease. Although the benefit may not be substantial in those with extrahepatic disease, it is plausible to treat these patients since the liver is typically the primary life-limiting factor.

Even in this cohort of chemorefractory patients, an objective response to ^90^Y therapy was seen in a large proportion of those patients who had follow-up data available. A significant biochemical response, CEA decrease by ≥50%, was seen in 41% of patients. Although the median survival was improved in those with a CEA response, this was not a significant difference. Jakobs et al. had reported a significant difference in survival for those with any decrease in CEA with a median survival of 19.1 months in responders versus 5.4 months in nonresponders [19]. Kennedy et al. have also reported a significant difference in survival between those who had an objective response, either biochemically or radiographically [12]. The lack of significance seen in our study may be due to the larger number of patients with more advanced disease in our sample.

When an objective radiographic response using RECIST criteria was analyzed, 12.9% of patients had a partial response and 64.5% of patients had stable disease. In other similar retrospective studies the partial response was 17–35.5%, stable disease was 55–61%, and progressive disease was 9.8–10% [12, 19]. There was not a significant difference in survival associated with a radiographic response. However, it should be noted that follow-up imaging was available for only 31 of our patients and thus limits objective radiographic response analysis in this study.

The use of ^90^Y is shown to be well tolerated in patients with advanced disease. The observed toxicities in this report were similar to those noted by others with fatigue, abdominal pain, and nausea the most frequent subjective complaints. The incidence of these complaints was less than previously reported. Sato et al. had reported the incidence of fatigue, abdominal pain, and nausea to be 56%, 26%, and 23%, respectively, compared to 22%, 16%, and 12% observed in this cohort [15]. There were grade 2 bilirubin toxicities, either early or late, seen in only 9 patients, and grade 3 and 4 toxicities were observed in only one patient each. The pretreatment inclusion criteria of a total bilirubin level of <2.0 is likely adequate to select patients with adequate hepatic reserve to undergo treatment. Although there was one incidence of an upper gastrointestinal bleed from esophageal varices; soon after treatment we did not observe the known complications of treatment-related peptic ulcers and pneumonitis. 

Our series has reinforced the findings of others. Mulcahy et al. reported their single-institution series of 72 patients using glass microspheres [26]. The treatment was well tolerated in these patients with self-limited treatment-related toxicities of fatigue, abdominal pain, and nausea, 61%, 25%, and 21%, respectively. Nine patients (12.6%) had grade 3 or 4 bilirubin toxicities. Using WHO criteria, a PR was noted in 40.3%, SD in 44.5%, and PD in 14.8%. The median time to PR was 4 months with a time to hepatic progression of 15.4 months. Survival analyses showed a median survival of 14.5 months from the time of initial treatment. Favorable predictors of survival were radiographic response, performance status, ≤25% tumor burden, and absence of extrahepatic disease. The median survival was not affected by the chemotherapy received prior to ^90^Y treatment. 

Several others have looked at the efficacy and safety of ^90^Y radioembolization combined with a radiosensitizing chemotherapy regimen as salvage therapy [27–29]. In patients who had failed previous oxaliplatin- and irinotecan-based therapy, in a series of 46 evaluable patients radiographic response using RECIST criteria was CR in 2%, PR in 22%, SD in 24%, and PD in 44% [27]. In another recent publication, Ricke et al. published their phase I study analyzing the efficacy and safety of combining systemic chemotherapy, using irinotecan, and ^90^Y treatment for second-line therapy [28]. The combination therapy had an acceptable toxicity profile, not reaching the maximal tolerated dose using up to 100 mg/m^2^ of irinotecan on days 1 and 8 of a 3-week cycle. A large proportion of patients had extrahepatic disease, 48%, and the site of first disease progression after treatment was extrahepatic in 57%. The efficacy was promising with either radiographically PR or SD in 87% of patients. The median progression free survival was 6.0 months and progression free survival in the liver was 9.2 months. In yet another study, Van den Eynde et al. performed a multicenter randomized controlled phase III trial to assess the addition of ^90^Y resin microspheres to continuous infusion of 5FU in 46 patients [29]. The median time to liver progression was significantly longer in patients receiving RE compared with 5FU alone, 5.5, and 2.1 months, respectively. The median survival was 7.4 months in the 5FU-only arm and 9.9 months in patients receiving 5FU plus ^90^Y. This data leads us to question if patients with liver dominant mCRC should have multimodality treatment with ^90^Y and systemic chemotherapy earlier in the treatment algorithm.

It should be noted that the administration of ^90^Y for mCRC is a complex process that requires a multidisciplinary team. The cost of this process can be substantial. However, we feel that this cost compares favorably to a course of adjuvant therapy with biological agents such as bevacizumab. The treatments are generally well tolerated with the goal of maintaining a high quality of life during therapy.

In conclusion, our report represents one of the larger single-institution reports on the safety and efficacy of ^90^Y for chemorefractory patients with advanced liver-dominant mCRC. We provide data to support the use of ^90^Y in the salvage setting for patients after failing the latest chemotherapeutic regimens even in the presence of limited extrahepatic disease. The use of ^90^Y to halt or slow the progression of the hepatic tumor burden in patients with a terminal disease process in an attempt to extend survival seems to be achievable. The major limitations of this study is that it is a retrospective study and although based in the setting of a single-institution the patient population was heterogeneous with most patients being referred to our tertiary center from other institutions. We present evidence to suggest that ^90^Y is an effective and safe treatment option in the salvage setting; however, there is still further validation required in the form of prospective trials which are ongoing. In order to convincingly support the benefit gained with locoregional therapy, progression free survival needs to be determined. Overall survival is often suboptimal to determine benefit since progression of disease will often prompt alternative treatments. Further work also needs to be done to evaluate whether ^90^Y may have benefit earlier in the treatment algorithm prior to the situation in which most, if not all other options are limited.

## Figures and Tables

**Table 1:** Patient characteristics of those treated with ^90^Y for liver-dominant metastatic colorectal cancer.

	*n *(%)	Median	Range
Age (years)		64	37–83
Male	35 (68.6)		
Female	16 (31.4)		
Time from diagnosis of metastases to 1st Rx (months)		23.2	1.3–99.9
Extrahepatic disease present	28 (58.3)		
Pulmonary nodules present	14 (28.5)		
Previous liver-directed therapy	16 (31.4)		
Previous liver resection	12 (23.5)		
Previous RFA	11 (21.5)		
Previous hepatic artery chemoinfusion	5 (9.8)		
Failed either bevacizumab or cetuximab*	33 (73.3)		
Failed both bevacizumab or cetuximab	9 (20.0)		
Failed capecitabine	14 (31.1)		

*Those receiving both bevacizumab and cetuximab also included.

**Table 2:** Treatment characteristics: Floxuridine (FUDR) was given with ^90^Y in some of our earlier patients. FUDR (5 mg/kg) was given just prior to administration of the ^90^Y microspheres.

	*n *(%)	Median	Range
Total number of treatments	90		
Patients with 1 treatment	20 (39.2)		
Patients with 2 treatments	27 (52.9)		
Patients with 4 treatments	4 (7.8)		
Radiation dose to target tissue per treatment (Gy)		44.4	12.9–67.2
Radiation activity per treatment (GBq)		0.89	0.16–2.20
FUDR with ^90^Y*			
Yes	17 (33.3)		
No	34 (66.7)		
Lung shunt (%)		3.3	0.4–11.5
Treatments terminated early due to embolic process	40 (44.4)		
Treatments with ≥80% of prescribed dose administered	67 (74.4)		

*Those who had prior hepatic artery chemoinfusion separate from the time of ^90^Y administration are not included in this group.

**Table 3:** Clinical endpoints: survival, biochemical, and radiologic response. Survival analyses to compare whether survival advantage was associated with studied variables was performed using log-rank analysis.

	*n *(%)	Median survival (months)	95% CI^1^
Died during follow-up	38 (74.5)		
Overall survival (months)		10.2	7.5–13.0
Gender	51 (100)		*P* = .64
M	35 (68.6)	10.6	3.4–17.8
F	16 (31.4)	10.2	0.0–22.0
Age	51 (100)		*P* = .18
<50	10 (20)	5.3	0.8–9.8
>50	41 (80)	8.2	1.8–19.4
FUDR given with ^90^Y			*P* = .72
Yes	17 (33.3)	17.0	6.7–27.3
No	34 (66.7)	8.2	4.2–12.3
Extrahepatic disease present	48 (100)		*P* = .07
Yes	28 (58.3)	6.7	2.7–10.6
No	20 (41.7)	17.0	6.4–27.6
CEA response^2^	41 (100)		*P* = .36
Yes	17 (41.5)	19.1	6.3–31.9
No	24 (42.9)	9.3	6.0–12.7
Radiographic Response^3^	31 (100)		*P* = .21
Progressive Disease	7 (22.6)	13.6	7.4–19.8
Stable Disease	20 (64.5)	9.3	6.9–11.8
Partial Response	4 (12.9)	21.5	13.7–29.1

^1^95% Confidence interval.

^2^CEA response defined as a reduction in CEA ≥50% of pretreatment value.

^3^RECIST criteria used to compare baseline measurement just prior to 1st treatment with radiologic response during 1–6 months follow-up imaging.

**Table 4:** Survival analysis of those who had received bevacizumab and/or cetuximab prior to treatment with ^90^Y.

	*n *(%)	Median Survival (months)	95% CI^1^
Failed cetuximab	44 (100)		*P* = .001
Yes	16 (36.4)	5.1	2.6–7.5
No	28 (63.6)	18.3	6.5–30.0
Failed bevacizumab	44 (100)		*P* = .36
Yes	17 (38.6)	8.2	5.0–11.4
No	27 (61.4)	17.0	6.7–27.3
Failed both bevacizumab and cetuximab	44 (100)		*P* = .001
Yes	9 (20)	5.2	2.9–7.4
No	35 (80)	13.6	4.9–22.2

^1^95% Confidence interval.
